# Acceptance and Use of Technology on Digital Learning Resource Utilization and Digital Literacy Among Chinese Engineering Students: A Longitudinal Study Based on the UTAUT2 Model

**DOI:** 10.3390/bs15060728

**Published:** 2025-05-24

**Authors:** Xinqiao Liu, Jingxuan Wang, Yunfeng Luo

**Affiliations:** 1 School of Education, Tianjin University, Tianjin 300350, Chinajingxuan_wang@tju.edu.cn (J.W.); 2 School of Public Administration, University of Electronic Science and Technology of China, Chengdu 611731, China

**Keywords:** engineering students, digital literacy, digital learning resources, behavioral intention, UTAUT2

## Abstract

With the rapid development of digital technology, the use of digital learning resources has become increasingly widespread and is now an integral part of higher education. However, there is a lack of research on engineering students’ behavioral intention to use digital resources and their digital literacy. This study, which is based on two waves of longitudinal survey data from 422 Chinese engineering students, employs the unified theory of acceptance and use of technology 2 (UTAUT2) model to systematically analyze the factors influencing engineering students’ behavioral intention to use digital learning resources and explore the longitudinal relationship between this intention and digital literacy. The results show that engineering students’ behavioral intention to use digital learning resources positively predicts their digital literacy. Effort expectancy, social influence, facilitating conditions, hedonic motivation, and habit are positively related to engineering students’ behavioral intention, whereas performance expectancy and price value do not have significant effects. The findings extend the application of the UTAUT2 model in the context of digital education and reveal a longitudinal link between the use of digital learning resources and digital literacy. This provides theoretical support and practical guidance for optimizing digital learning environments, enhancing digital literacy in engineering students, and improving the design of teaching resources in higher education, contributing to the development of engineering education in China in the digital age.

## 1. Introduction

In the age of digital intelligence, the way knowledge is acquired has entered a new phase. Students’ learning methods have shifted from traditional printed resources to digital learning resources (Alphonce & Mwantimwa, 2019), indicating that digital transformation in higher education is a key direction for future educational development. Digital learning resources, such as massive open online courses (MOOCs), learning management systems (LMSs), and intelligent learning assistants, are gradually being integrated into students’ daily academic routines, becoming the primary pathway for acquiring knowledge (Anshari et al., 2016). These resources not only reduce the cost of the learning infrastructure but also facilitate the digitalization of course content. Students can engage in immersive learning and interaction through smart devices, contribute resources and collaborate with teachers via online platforms, creating a more convenient and interactive learning experience (Pham et al., 2019; Kim et al., 2019). Digital learning resources primarily support students’ self-directed learning, significantly enhancing their independent learning, information retrieval, teamwork, and digital skills (Ramírez-Donoso et al., 2023; Camilleri & Camilleri, 2017). According to data from the European Union, approximately one-quarter to one-third of students in Europe attend schools supported by digital technologies. The South Korean government is also investing in the development of e-learning environments on campuses, providing students with more intellectually engaging learning experiences (Kim et al., 2019). Progress in e-learning environments across universities worldwide has contributed to improving students’ academic success. In the United States, 43% of students use digital media in classrooms daily (Küsel et al., 2020), whereas students in Spain and Italy frequently use video and audiovisual materials as digital learning resources (Tejedor et al., 2020). Digital learning resources are now widely used among university students globally. As a result, understanding the factors that influence students’ behavioral intentions to engage with these resources, as well as focusing on enhancing their digital literacy, has become a critical task for the future of higher education.

Under the wave of educational digital transformation, students need to learn how to independently manage their learning in a digital environment. Digital literacy has emerged as a key ability for university students to thrive in blended learning settings (Tang & Chaw, 2016). Initially, Gilster (1997) defined digital literacy as “the ability to understand and use information from a wide range of sources, presented in various formats via computers.” UNESCO defines digital literacy as the ability to safely and appropriately access, manage, understand, integrate, communicate, evaluate, and create information via digital devices and network technologies to engage in economic and social life. Academics define digital literacy as the ability to use and interpret information from various digital sources (Gilster, 1997; Lankshear & Knobel, 2015), which includes not only technical operational skills but also cognitive, emotional, and social competencies. These skills are crucial for effective learning in a digital environment. Digital literacy supports lifelong self-learning for students (Anthonysamy et al., 2020). University students, as digital natives, have been exposed to and have used digital technologies from an early age, making them highly familiar with technology. However, despite their familiarity with technology, they may still lack digital literacy, which involves the ability to effectively use, evaluate, and utilize digital learning resources for academic and practical purposes. The gap between “exposure” and the formation of digital literacy makes it difficult for them to efficiently filter learning materials, develop self-directed learning strategies, or resist the distractions of information overload, directly affecting their performance in digital learning (Kennedy & Fox, 2013; Muasyaroh & Royanto, 2024; X. Liu et al., 2023). Therefore, focusing on university students’ digital literacy is not only the foundation of digital education but also the key to helping students develop lifelong learning skills in hybrid educational environments.

The characteristics of academic disciplines significantly shape students’ digital resource usage patterns, with different disciplines having distinct approaches. For example, law students use the Lovdata system for legal studies, medical students utilize e-learning platforms for medical education, and humanities students collect materials through national library digital corpora (Bygstad et al., 2022). In comparison, engineering students face the dual challenges of technology intensity and system complexity. They must gain proficiency in essential technologies while continuously adapting to rapidly changing digital tools and technological ecosystems. Engineering students are at the forefront of digital transformation. Currently, the time engineering students spend on digital learning resources tends to be fragmented, with some encountering cognitive overload, a lack of digital ethics awareness, and insufficient digital literacy, all of which affect their behavioral intentions to use these resources (Miranda et al., 2021; Skulmowski & Xu, 2022; Ramírez-Donoso et al., 2023; Gao et al., 2023; Anthonysamy et al., 2020). As a key group for cultivating digital talent, the industrial 5.0 era presents greater demands on digital literacy and the use of digital learning resources by engineering students (Broo et al., 2022; Motyl et al., 2017). Exploring engineering students’ behavioral intentions and digital literacy will help them adapt to the digital education environment, promote innovation in engineering talent development, and accelerate the development of the new era’s technological revolution.

This study, which is based on the unified theory of acceptance and use of technology 2 (UTAUT2) model and longitudinal tracking survey data, explores the predictive relationship between Chinese engineering students’ use of digital learning resources and their digital literacy, as well as the factors influencing their behavioral intention to use these resources. Building on previous research that has demonstrated the applicability of the UTAUT2 model to student populations (Azizi et al., 2020; Nikolopoulou et al., 2020). In this study, UTAUT2 model not only reveals multiple factors influencing engineering students’ use of digital learning resources but also explains their technological adaptability in digital learning environments. Additionally, studying the longitudinal relationship between the behavioral intention to use digital learning resources and digital literacy is important for optimizing digital teaching strategies in engineering education and enhancing the quality of engineering education in China.

## 2. Literature Review

### 2.1. Relationship Between Behavioral Intention to Use Digital Learning Resources and Digital Literacy

Previous studies have shown a positive relationship between behavioral intention to use digital learning resources and digital literacy (Bygstad et al., 2022; T. L. H. Le et al., 2022; Lim & Newby, 2021; Ting, 2015). For students, digital literacy includes effectively planning and managing available information, avoiding cognitive overload, and staying on track during searches while also monitoring progress toward achieving learning goals (Bråten et al., 2011; Gerjets et al., 2008). In the process of using digital learning resources, students can develop various aspects of digital literacy, including visual information processing, information evaluation, and content creation (Blau et al., 2020). When students show a high level of interest in learning and applying new technologies, their behavioral intention to use digital learning resources tends to be greater. This motivation can enhance their ability to learn new technologies and improve self-regulation, thus increasing their digital literacy (Lilian, 2022; Blau et al., 2020). When students extensively use digital learning resources for educational purposes—such as searching for references, using translation software, or engaging with online learning platforms—it can increase their involvement in learning, which in turn helps develop their digital skills and, to some extent, improve their digital literacy (Ben Youssef et al., 2022; Calafiore & Damianov, 2011). A cross-sectional study by Vodă et al. (2022) of 259 students in Romania revealed that students who used digital learning resources had higher levels of digital literacy in areas such as information processing, problem solving, and technical skills than those who did not use such resources. B. Le et al. (2022) studied 234 students in Australia enrolled in STEM foundational courses at a research university and reported that some students compensated for their lack of digital literacy by using digital learning resources more frequently, which resulted in better performance on certain assessments. Makinde and Okoye (2024), via hierarchical multiple regression analysis of 304 students, reported that the behavioral intention to use digital learning resources is the best predictor of actual resource use.

Some scholars argue that digital literacy positively influences students’ behavioral intention to use digital learning resources. Students with greater digital literacy tend to possess strong information retrieval skills, information processing abilities, and learning focus, making them more willing to use digital learning resources to achieve their learning outcomes (Reddy et al., 2021, 2023; Yustika & Iswati, 2020). Greene et al. (2014) used the think-aloud method in a study with 20 undergraduates in the United States and reported that self-regulated learning and cognitive awareness—key components of digital literacy—had a significant positive effect on the use of digital learning resources. Jang et al. (2021), through structural equation modeling, studied young adults (aged 20–30) in South Korea and Finland and reported that digital literacy positively influenced behavioral intention through the mediating roles of habits and performance expectancy. Hsu et al. (2019) conducted a mixed-methods case study with 32 elementary school students in Taiwan and reported that course designs involving digital learning resources significantly improved students’ digital literacy in areas such as information management, collaboration, communication, sharing, and creativity. However, digital literacy related to ethics and responsibility was not enhanced.

To date, many studies have explored the relationship between behavioral intention to use digital learning resources and digital literacy, but longitudinal research is lacking. Since digital literacy is not a static skill level at a single point in time but rather improves as students continue to use digital learning resources, more research is needed to better understand the predictive relationship between behavioral intention to use digital learning resources and digital literacy. On the basis of the findings of previous studies, we propose Hypothesis 1.

**Hypothesis** **1.** *Engineering students’ behavioral intention to use digital learning resources positively predicts their digital literacy.*

### 2.2. Factors Influencing University Students’ Behavioral Intentions to Use Digital Learning Resources

The factors influencing students’ behavioral intentions to use digital learning resources are complex and diverse. Scholars tend to apply various theoretical frameworks to understand behavioral intentions in different contexts. Sayaf et al. (2021), on the basis of the technology acceptance model (TAM) and social cognitive theory (SCT), investigated students from two universities in Saudi Arabia and reported that computer self-efficacy, perceived ease of use, and perceived usefulness are key factors influencing students’ continued use of digital technologies. Songkram et al. (2023) studied 1406 K-12 students across Thailand via TAM and structural equation modeling. They reported that the main factors affecting students’ use of digital learning platforms include their attitudes toward the platform, their perceived usefulness, and their perceived ease of use. Students’ use of digital learning resources is also influenced by subjective norms, attitudes, and other factors (Bai & Jiang, 2024; Potnis et al., 2018). Some scholars have used the UTAUT model for analysis, and compared with the TAM, the UTAUT model is more comprehensive (Venkatesh et al., 2003). In a study on Taiwanese university students’ behavioral intentions to use English online learning sites, Mohammadyari and Singh (2015) reported that performance expectancy, effort expectancy, and social influence positively affected students’ behavioral intentions to use digital learning resources. Facilitating conditions, related to students’ motivation to use digital learning resources and the accessibility and availability of innovative resources in the educational environment, also play crucial roles (Sardone & Devlin-Scherer, 2010). Facilitating conditions are positive influencers of behavioral intentions (Saad et al., 2021) and include factors such as a digitally skilled faculty team (Hoskins & Crick, 2010; Rafi et al., 2019), the technological infrastructure of schools, and high-quality digital learning resources (Alphonce & Mwantimwa, 2019; Miranda et al., 2021). The quality of electronic services and information is vital to students’ e-learning experiences, and good facilitating conditions can significantly enhance their behavioral intentions to use digital learning resources (Shehzadi et al., 2021).

Avci (2022), utilizing the UTAUT2 model, investigated teachers’ behavioral intention to use digital learning resources and reported that hedonic motivation, performance expectancy, and habits had a positive effect on behavioral intention, whereas effort expectancy, social influence, facilitating conditions, and price value did not have a significant effect. Some studies suggest that performance expectancy, social influence, facilitating conditions, and price value contribute to teachers’ adoption of MOOCs, but effort expectancy and hedonic motivation do not drive teachers’ adoption of MOOCs (Tseng et al., 2022). J. Liu et al. (2025), in their study of teachers’ behavioral intention to use digital resources with the UTAUT2 model, did not include the price value variable, as they argued that teachers generally do not consider price value when using digital resources. Their findings showed that performance expectancy, social influence, hedonic motivation, and habits had significant positive effects on teachers’ behavioral intentions to use digital resources, with habits having the strongest effect. Effort expectancy and facilitating conditions did not have significant effects on behavioral intentions. A study on medical students’ behavioral intentions to use blended learning, which was based on the UTAUT2 model, revealed that performance expectancy, effort expectancy, social influence, facilitating conditions, hedonic motivation, price value, and habits all had significant positive impacts on students’ behavioral intentions (Azizi et al., 2020). Most studies suggest that performance expectancy positively influences behavioral intentions (Abdekhoda et al., 2016; Tarhini et al., 2017; Wijaya et al., 2025) and that effort expectancy positively influences behavioral intentions (Tarhini et al., 2017; Tam et al., 2020). Social influence positively affects behavioral intention (Šumak & Šorgo, 2016; Risman & Budiarti, 2023), and facilitating conditions are a positive factor for behavioral intention (Dahri et al., 2021; Venkatesh et al., 2016). Hedonic motivation positively influences behavioral intentions (Nikolopoulou et al., 2020; Garg, 2022), and price value positively affects behavioral intentions (Rezeki et al., 2023). Habit also positively influences behavioral intentions (Zhou et al., 2022).

However, there are contradictions in the research findings regarding the factors that influence the behavioral intention to use digital learning resources in different research contexts. More studies using the UTAUT2 model are needed, as are further explorations of the factors influencing specific student groups’ behavioral intentions to use digital learning resources. On the basis of the above research, we propose Hypotheses 2–8.

**Hypothesis** **2.** *Performance expectancy has a positive influence on engineering students’ behavioral intentions to use digital learning resources.*

**Hypothesis** **3.** *Effort expectancy has a positive influence on engineering students’ behavioral intentions to use digital learning resources.*

**Hypothesis** **4.** *Social influence has a positive influence on engineering students’ behavioral intentions to use digital learning resources.*

**Hypothesis** **5.** *Facilitating conditions positively influence engineering students’ behavioral intentions to use digital learning resources.*

**Hypothesis** **6.** *Hedonic motivation has a positive influence on engineering students’ behavioral intention to use digital learning resources.*

**Hypothesis** **7.** *Price value has a positive influence on engineering students’ behavioral intention to use digital learning resources.*

**Hypothesis** **8.** *Habit has a positive influence on engineering students’ behavioral intentions to use digital learning resources.*

## 3. Theoretical Framework and Research Objectives

### 3.1. Theoretical Framework

This study is grounded in the Unified Theory of Acceptance and Use of Technology 2 (UTAUT2). The original UTAUT model, developed by Venkatesh et al. (2003), is primarily used for employee groups. It integrates key concepts from eight major theories of technology acceptance: the Theory of Reasoned Action (TRA), the Technology Acceptance Model (TAM), the Motivation Model (MM), the Theory of Planned Behavior (TPB), the combined TAM and TPB model, the Model of PC Utilization (MPCU), the Innovation Diffusion Theory (IDT), and Social Cognitive Theory (SCT). UTAUT provides a comprehensive framework for evaluating the relationships between various factors affecting an individual’s acceptance of technology (Huang et al., 2013). The UTAUT model includes four key constructs: performance expectancy, effort expectancy, social influence, and facilitating conditions. Performance expectancy refers to the degree to which an individual believes that using a technology will enhance their work performance. A high level of performance expectancy indicates a strong belief that the technology will improve productivity or learning outcomes. Effort expectancy refers to the degree to which an individual perceives a technology as easy to use. A high level of effort expectancy indicates that the individual believes using the technology will require minimal effort. Social influence refers to the extent to which individuals believe that others think they should use the technology. Facilitating conditions refer to the perceived availability of organizational and technical infrastructure to support technology use, such as access to necessary resources, training, or support systems (Venkatesh et al., 2003).

**Figure 1:**
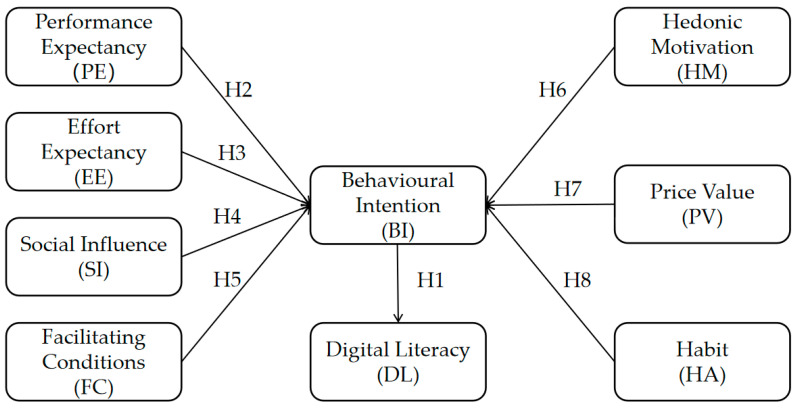
Research model based on the expanded version of UTAUT2.

Venkatesh et al. (2012) expanded the original UTAUT model by introducing UTAUT2, which is primarily used to understand consumers’ technology acceptance. It includes three additional constructs: hedonic motivation, price value, and habit. Hedonic motivation refers to the enjoyment or pleasure derived from using the technology (Venkatesh et al., 2012). Price value is defined as an individual’s evaluation of the perceived benefits of using the system relative to the monetary cost incurred for its adoption (Chao, 2019). A high price value indicates that the perceived benefits outweigh the monetary cost. Habit is the degree to which individuals tend to automatically perform behavior due to repeated use without conscious intention (Limayem et al., 2007). A strong habit implies that the behavior has become routine and requires little deliberate thought or conscious decision-making. The theoretical framework for this study, which is based on UTAUT2, is illustrated in Figure 1.

### 3.2. Research Objectives

There is little research on engineering students, a crucial demographic, particularly concerning their behavioral intention to use digital learning resources and the development of their digital literacy. Given the technology-intensive nature of engineering disciplines, digital learning resource usage patterns and digital literacy development among engineering students are unique, warranting focused research in this area. Furthermore, most existing studies have relied on cross-sectional data to analyze the relationship between behavioral intention and digital literacy, with a lack of longitudinal data, making it difficult to observe how these variables change over time.

This study focuses on Chinese engineering students and aims to enhance the digital education environment for this group, thereby promoting the development of engineering education in China. This study innovatively incorporates longitudinal data into the UTAUT2 model and uses partial least squares structural equation modeling (PLS-SEM) to analyze how the first wave of students’ behavioral intention to use digital learning resources influences their digital literacy level in the second wave. This approach strengthens the predictive relationship between the variables, addressing the limitations of previous research.

In addition, the UTAUT2 model provides a more comprehensive understanding of the key factors influencing the behavioral intention to use digital learning resources. The findings of this research are expected to improve engineering students’ willingness to use digital learning resources and facilitate their integration into the digital education environment.

## 4. Methodology

### 4.1. Participants

The study sample consists of engineering students from a prestigious university in China. Data were collected via self-report questionnaires. A total of 422 engineering students participated in a longitudinal survey conducted in two waves, in November 2023 and May 2024. The sample includes 143 first-year students, 188 second-year students, and 89 third-year students. Among the participants, 184 reported using digital learning resources almost every day, 133 used them 3–4 times per week, and 91 used them 1–2 times per week. All participants voluntarily agreed to participate in this study and signed an informed consent form before the study commenced. This research was supported by the university’s administrative department.

### 4.2. Measurement Variables

This study was designed on the basis of the UTAUT2 framework. Performance expectancy (PE) and effort expectancy (EE) were each measured via four items, whereas social influence (SI), facilitating conditions (FC), hedonic motivation (HM), price value (PV), habit (HA), and behavioral intention (BI) were each measured via three items. Digital literacy (DL) was measured via 12 items.

The scales for Performance Expectancy, Effort Expectancy, Social Influence, Facilitating Conditions, Hedonic Motivation, Price Value, Habit, and Behavioral Intention were adapted from the works of Venkatesh et al. (2003, 2012), Chao (2019), and Limayem et al. (2007). These were measured via a 5-point Likert scale ranging from 1 (strongly disagree) to 5 (strongly agree). The measurement items for Performance Expectancy included statements such as “Using digital learning resources helps me complete learning tasks faster”. The items for Effort Expectancy included “I can easily obtain the information I want through digital learning resources”. Social Influence was measured with items such as “If my teacher or classmates recommend using digital learning resources, I would be willing to use them”. Facilitating Conditions was assessed using items such as “I have the necessary equipment and network to use digital learning resources”. The scale for Hedonic Motivation included items like “Using digital learning resources is fun”. Items for Price Value included “The time cost of using digital learning resources is worth it”. Habit was measured using items such as “I am accustomed to using digital learning resources”. Behavioral Intention included items such as “If conditions allow, I am willing to use digital learning resources”. Digital literacy was designed on the basis of the 12 graduation requirements for Chinese engineering students as outlined by the China Engineering Education Accreditation Association. This scale was collected during the second wave of the survey via a 7-point Likert scale ranging from 1 (strongly disagree) to 7 (strongly agree). The measurement items for Digital Literacy included statements such as “I can use digital resources, technologies, or tools to identify and assess key issues in complex problems”. and “I can comply with laws and regulations related to cybersecurity, data security, etc., and use digital technologies, resources, and tools in a reasonable and lawful manner.”

### 4.3. Data Analysis

Data cleaning was performed via Stata 17, and partial least squares structural equation modeling (PLS-SEM) was used as the primary method for data analysis, which was conducted with SmartPLS 4.1 software. PLS-SEM is suitable for small sample sizes and nonnormal data and is advantageous for exploring complex relationships among latent variables and conducting exploratory research (Hair et al., 2011). The following steps were taken in the data analysis: (1) We examined the internal consistency and convergent validity of the measurement scales. Cronbach’s alpha and composite reliability (CR) were used as indicators of internal consistency. Both values should exceed 0.7 to ensure reliable internal consistency. Convergent validity was assessed via factor loadings and average variance extracted (AVE). Factor loadings should exceed 0.7, and the AVE value should be greater than 0.5 to demonstrate satisfactory convergent validity (Hair et al., 2019). (2) Discriminant validity was assessed via the Fornell and Larcker criterion. For discriminant validity to be present, the AVE of each latent variable should be greater than the squared correlation between that latent variable and any other latent variables (Fornell & Larcker, 1981). (3) We estimated the path coefficients and their significance to quantify the effects of the independent variables on the dependent variables. The bootstrapping algorithm was applied with a subsample size of 5000 and a significance level of 0.05. The path coefficients (β) and significance values (*p*) were obtained. Additionally, Cohen’s f^2^ values of 0.02, 0.15, and 0.35 represent small, medium, and large effect sizes, respectively (Cohen, 1988).

## 5. Results

### 5.1. Measurement Results of the Scales

As shown in Table 1, the Cronbach’s alpha values for the latent variables in the model range from 0.843 to 0.889, and the CR values range from 0.850 to 0.900, all of which are significantly greater than 0.7, indicating high internal consistency and reliability of the scales. The factor loadings for all the items range from 0.734 to 0.937, which are all greater than 0.7, indicating strong correlations between the measurement items and their corresponding latent variables, effectively reflecting the concepts of the latent variables. The AVE values range from 0.697 to 0.818, all of which are above 0.5, indicating that each latent variable explains a large portion of the variance of its measurement items, demonstrating ideal convergent validity. In addition, the students’ digital literacy was measured by the average score of 12 survey questions, with higher scores indicating higher digital literacy. The Cronbach’s alpha for digital literacy is 0.9632, indicating good internal consistency of the model.

### 5.2. Fornell–Larcker Criterion Test

Discriminant validity refers to the ability of the scale to distinguish between different constructs, avoiding overlap between the measurement items of the same construct. This was tested via the Fornell–Larcker criterion, where the diagonal values should be greater than the squared correlations of the corresponding rows or columns. As shown in Table 2, the square roots of the AVE for all latent variables are greater than their correlations with other latent variables, further indicating that each latent variable explains most of the variance in its measurement items, whereas the correlations with other latent variables remain within reasonable limits. This confirms that the latent variables are conceptually independent and that the model has good discriminant validity.

### 5.3. PLS–SEM Results Analysis

**Figure 2:**
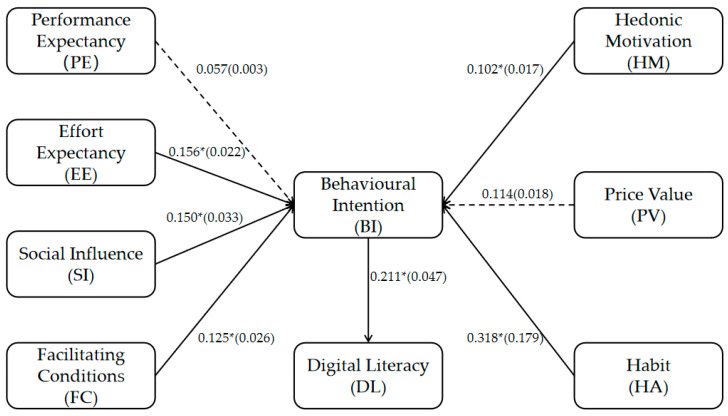
Path coefficient analysis results. Note: * Indicates *p* < 0.05. The value of f^2^ is in parentheses. The solid lines indicate statistically significant path coefficients, whereas the dashed lines represent paths that do not have statistical significance.

The results of the path analysis are shown in Figure 2. Among the eight hypotheses proposed, six received statistical support, as shown in Table 3. Specifically, performance expectancy (PE) (β = 0.057, *p* = 0.361 > 0.05) had no significant effect on behavioral intention (BI), with f^2^ = 0.003, indicating a very small effect of PE on BI. Effort expectancy (EE) (β = 0.156, *p* = 0.025 < 0.05) had a significant effect on BI, with f^2^ = 0.022, indicating a small effect of EE on BI. Social influence (SI) (β = 0.150, *p* = 0.025 < 0.05) had a significant effect on BI, with f^2^ = 0.033, indicating a small effect of SI on BI. Facilitating conditions (FC) (β = 0.125, *p* = 0.042 < 0.05) had a significant effect on BI, with f^2^ = 0.026, indicating a small effect of FC on BI. Hedonic motivation (HM) (β = 0.102, *p* = 0.044 < 0.05) had a significant effect on BI, with f^2^ = 0.017, indicating a very small effect of HM on BI. The price value (PV) (β = 0.114, *p* = 0.070 > 0.05) had no significant effect on BI, with f^2^ = 0.018, indicating a very small effect of PV on BI. Habit (HA) (β = 0.318, *p* = 0.000 < 0.05) had a significant effect on BI, with f^2^ = 0.179, indicating a medium effect of HA on BI. BI (β = 0.211, *p* = 0.000 < 0.05) had a significant effect on digital literacy (DL), with f^2^ = 0.047, indicating a small effect of BI on DL. The model demonstrates strong explanatory power for BI, accounting for 74% of its variance. In contrast, the explanatory power for DL is relatively modest. The Q^2^ values for both BI and DL are greater than 0, indicating that the model has acceptable predictive relevance for both constructs. Overall, the behavioral intention of engineering students to use digital learning resources in the first wave positively predicts their digital literacy in the second wave. Effort expectancy, social influence, facilitating conditions, hedonic motivation, and habit are positive influencing factors of engineering students’ behavioral intention to use digital learning resources, while performance expectancy and price value have no significant effect on their intention to use these resources.

## 6. Discussion

This study, focusing on Chinese engineering students, uses the UTAUT2 and PLS-SEM models to analyze the factors influencing their behavioral intention to use digital learning resources and the longitudinal predictive relationship between behavioral intention and digital literacy.

First, engineering students’ behavioral intention to use digital learning resources positively predicts their digital literacy level. This indicates that the stronger engineering students’ intention to use digital learning resources, the higher their digital literacy development, consistent with previous research findings (Bygstad et al., 2022; T. L. H. Le et al., 2022). Behavioral intention reflects students’ motivation to use digital learning resources. Students with higher behavioral intention are more likely to actively explore and utilize digital tools due to their greater interest in and demand for such technologies. This motivation encourages them to engage more proactively and deeply with new tools, which in turn enhances their ability to use and interpret information from various digital sources, ultimately improving their digital literacy (Lilian, 2022; Blau et al., 2020). In particular, engineering students with high behavioral intention tend to use specialized engineering software, programming tools, and online learning platforms to meet academic requirements. Through continued use and deepening understanding of these resources, they improve their information filtering abilities, technical proficiency, and problem-solving skills, which in turn contributes to the development of higher levels of digital literacy (Vodă et al., 2022).

Second, effort expectancy, social influence, facilitating conditions, hedonic motivation, and habits are positive factors influencing engineering students’ behavioral intention to use digital learning resources. Effort expectancy positively influences engineering students’ behavioral intention to use digital learning resources, which is consistent with previous research (Tarhini et al., 2017; Tam et al., 2020; Mohammadyari & Singh, 2015; Azizi et al., 2020). The ease of use of digital learning resources (such as intuitive interfaces and effective guidance) enhances engineering students’ effort expectancy by reducing the difficulty of use, thereby increasing their willingness to use them (Zhu & Yang, 2023; Skulmowski & Xu, 2022). Social influence positively affects engineering students’ behavioral intention to use digital learning resources, which is consistent with the findings of previous studies (Šumak & Šorgo, 2016; Risman & Budiarti, 2023; Mohammadyari & Singh, 2015; Azizi et al., 2020; Tseng et al., 2022; J. Liu et al., 2025). This suggests that in an interactive academic environment, the influence of classmates, teachers, and peers positively affects their use of digital learning resources. Facilitating conditions are also positive factors influencing engineering students’ behavioral intention to use digital learning resources, which is consistent with previous research (Dahri et al., 2021; Venkatesh et al., 2016; Sardone & Devlin-Scherer, 2010; Saad et al., 2021; Tseng et al., 2022; Azizi et al., 2020). This finding indicates that external conditions such as technological infrastructure and the network environment of digital learning resources in universities directly influence engineering students’ behavioral intention to use them. This suggests that providing convenient and user-friendly digital learning resources can enhance their intention to use them (Sardone & Devlin-Scherer, 2010). Hedonic motivation is a positive factor influencing engineering students’ behavioral intention to use digital learning resources, which is consistent with previous research (Nikolopoulou et al., 2020; Garg, 2022; Avci, 2022; J. Liu et al., 2025; Azizi et al., 2020). For engineering students, if digital learning resources offer fun or gamified experiences, their willingness to use these resources will increase, as they derive enjoyment from using them. Habit positively influences engineering students’ behavioral intention to use digital learning resources, which is consistent with the findings of previous studies (Zhou et al., 2022; Avci, 2022; J. Liu et al., 2025; Azizi et al., 2020). This study found that habit has the strongest impact on engineering students’ behavioral intention to use digital learning resources, with its influence being approximately twice that of effort expectancy and social influence. This indicates that the use of digital learning resources has been deeply integrated into the learning habits of engineering students, which aligns closely with the tool-embedded characteristics of engineering education.

Third, performance expectancy and price value do not have a significant effect on engineering students’ behavioral intention to use digital learning resources. In the research data, the influence of performance expectancy and price value on behavioral intention is minimal, and their actual effect is very limited, making them nonmajor factors and leading to insignificant results. Performance expectancy does not significantly affect engineering students’ behavioral intention to use digital learning resources, which is inconsistent with previous research (Mohammadyari & Singh, 2015; Abdekhoda et al., 2016; Tarhini et al., 2017; Avci, 2022; Tseng et al., 2022; Wijaya et al., 2025; J. Liu et al., 2025; Azizi et al., 2020). Performance expectancy typically refers to the expectation that digital learning resources can improve learning efficiency and simplify the learning process. However, engineering students are more concerned with whether digital learning resources meet specific task requirements rather than performance itself. Many engineering courses already have deeply integrated digital learning resources, and engineering students view them as essential learning tools. Therefore, performance improvement has less impact on their intention to use them, and their use tends to become routine and habitual. They are more likely to be driven by other factors rather than by the expectation of performance enhancement in digital learning resources. Price value does not significantly affect engineering students’ behavioral intention to use digital learning resources, which is consistent with Avci (2022) but inconsistent with other studies (Rezeki et al., 2023; Azizi et al., 2020). The reason for this may be that digital learning resources, such as course platforms and databases, are often provided free of charge by universities, so students perceive the monetary cost as nearly zero. Furthermore, engineering students are more focused on the alignment and practicality of the resources with their course requirements, rather than on evaluating the perceived benefits in relation to the monetary cost. Additionally, some digital learning resources required for specific majors are indispensable for engineering students, making them essential for their studies. As a result, price value is no longer a major factor influencing their intention to use these resources.

## 7. Limitations

This study has three limitations. First, the measurement of factors influencing engineering students’ behavioral intention to use digital learning resources and digital literacy relies on self-reported data, which may lead to measurement errors. Second, the research results are only applicable to Chinese engineering students, and the situation may differ for students from other countries. Third, the explanatory power of BI on DL is relatively limited, suggesting the need to include additional predictors in future studies to better understand the development of digital literacy. Fourth, future studies may consider including more moderating variables to analyze their impact on engineering students’ behavioral intention to use digital learning resources and digital literacy.

## 8. Conclusions

First, engineering students’ behavioral intention to use digital learning resources positively predicts their digital literacy.

Second, effort expectancy, social influence, facilitating conditions, hedonic motivation, and habits are positive factors influencing engineering students’ behavioral intention to use digital learning resources. Among these, habit has the strongest impact, with its influence being approximately twice that of effort expectancy and social influence.

Third, performance expectancy and price value do not significantly affect engineering students’ behavioral intention to use digital learning resources.

Therefore, universities should focus on cultivating engineering students’ habits of using digital learning resources to enhance their intention to use and improve learning outcomes. Educational design should pay more attention to hedonic motivation and facilitating conditions, providing students with more enjoyable and user-friendly learning tools and platforms, thus increasing their engagement and digital literacy. The findings of this study can provide valuable insights for higher education institutions in different countries and regions, further promoting the global digital transformation of higher education.

## Figures and Tables

**Table 1:** Measurement model evaluation results.

Variable	Factor Load Range	Cronbach’s Alpha	Composite Reliability	AVE
PE	0.734–0.896	0.867	0.869	0.718
EE	0.806–0.870	0.856	0.862	0.697
SI	0.835–0.913	0.845	0.850	0.765
FC	0.865–0.889	0.852	0.862	0.770
HM	0.853–0.931	0.888	0.900	0.817
PV	0.821–0.905	0.843	0.854	0.762
HA	0.903–0.905	0.889	0.891	0.818
BI	0.855–0.937	0.880	0.883	0.807

Note: PE = performance expectancy; EE = effort expectancy; SI = social influence; FC = facilitating conditions; HM = hedonic motivation; PV = price value; HA = habit; BI = behavioral intention.

**Table 2:** Fornell–Larcker criterion.

	PE	EE	SI	FC	HM	PV	HA	BI	DL
PE	0.847								
EE	0.818	0.835							
SI	0.732	0.703	0.874						
FC	0.668	0.719	0.608	0.878					
HM	0.657	0.681	0.625	0.511	0.904				
PV	0.741	0.707	0.672	0.628	0.670	0.873			
HA	0.615	0.683	0.616	0.537	0.615	0.622	0.904		
BI	0.725	0.765	0.713	0.661	0.675	0.712	0.753	0.898	
DL	0.286	0.307	0.240	0.240	0.234	0.195	0.245	0.211	1

Note: PE = performance expectancy; EE = effort expectancy; SI = social influence; FC = facilitating conditions; HM = hedonic motivation; PV = price value; HA = habit; BI = behavioral intention; DL = digital literacy.

**Table 3:** Acceptance and rejection of hypotheses.

Hypotheses	Acceptance	Rejection
Hypothesis 1	Yes	
Hypothesis 2		Yes
Hypothesis 3	Yes	
Hypothesis 4	Yes	
Hypothesis 5	Yes	
Hypothesis 6	Yes	
Hypothesis 7		Yes
Hypothesis 8	Yes	

## Data Availability

Data will be made available upon reasonable request.

## Abbreviations

The following abbreviations are used in this manuscript.

UTAUT2	Unified Theory of Acceptance and Use of Technology 2
PLS-SEM	Partial Least Squares Structural Equation Modeling
PE	Performance Expectancy
EE	Effort Expectancy
SI	Social Influence
FC	Facilitating Conditions
HM	Hedonic Motivation
PV	Price Value
HA	Habit
BI	Behavioral Intention
DL	Digital Literacy

## References

[B1-behavsci-15-00728] Abdekhoda M., Dehnad A., Mirsaeed S. J. G., Gavgani V. Z. (2016). Factors influencing the adoption of E-learning in Tabriz University of Medical Sciences. Medical Journal of the Islamic Republic of Iran.

[B2-behavsci-15-00728] Alphonce S., Mwantimwa K. (2019). Students’ use of digital learning resources: Diversity, motivations and challenges. Information and Learning Sciences.

[B3-behavsci-15-00728] Anshari M., Alas Y., Guan L. S. (2016). Developing online learning resources: Big data, social networks, and cloud computing to support pervasive knowledge. Education and Information Technologies.

[B4-behavsci-15-00728] Anthonysamy L., Koo A. C., Hew S. H. (2020). Self-regulated learning strategies in higher education: Fostering digital literacy for sustainable lifelong learning. Education and Information Technologies.

[B5-behavsci-15-00728] Avci S. (2022). Examining the factors affecting teachers’ use of digital learning resources with UTAUT2. Malaysian Online Journal of Educational Technology.

[B6-behavsci-15-00728] Azizi S. M., Roozbahani N., Khatony A. (2020). Factors affecting the acceptance of blended learning in medical education: Application of UTAUT2 model. BMC Medical Education.

[B7-behavsci-15-00728] Bai Y. Q., Jiang J. W. (2024). Meta-analysis of factors affecting the use of digital learning resources. Interactive Learning Environments.

[B8-behavsci-15-00728] Ben Youssef A., Dahmani M., Ragni L. (2022). ICT use, digital skills and students’ academic performance: Exploring the digital divide. Information.

[B9-behavsci-15-00728] Blau I., Shamir-Inbal T., Avdiel O. (2020). How does the pedagogical design of a technology-enhanced collaborative academic course promote digital literacies, self-regulation, and perceived learning of students?. The Internet and Higher Education.

[B10-behavsci-15-00728] Bråten I., Britt M. A., Strømsø H. I., Rouet J. F. (2011). The role of epistemic beliefs in the comprehension of multiple expository texts: Toward an integrated model. Educational Psychologist.

[B11-behavsci-15-00728] Broo D. G., Kaynak O., Sait S. M. (2022). Rethinking engineering education at the age of industry 5.0. Journal of Industrial Information Integration.

[B12-behavsci-15-00728] Bygstad B., Øvrelid E., Ludvigsen S., Dæhlen M. (2022). From dual digitalization to digital learning space: Exploring the digital transformation of higher education. Computers & Education.

[B13-behavsci-15-00728] Calafiore P., Damianov D. S. (2011). The effect of time spent online on student achievement in online economics and finance courses. The Journal of Economic Education.

[B14-behavsci-15-00728] Camilleri M. A., Camilleri A. C. (2017). Digital learning resources and ubiquitous technologies in education. Technology, Knowledge and Learning.

[B15-behavsci-15-00728] Chao C. M. (2019). Factors determining the behavioral intention to use mobile learning: An application and extension of the UTAUT model. Frontiers in psychology.

[B16-behavsci-15-00728] Cohen J. (1988). Statistical power analysis for the behavioral sciences.

[B17-behavsci-15-00728] Dahri N. A., Vighio M. S., Bather J. D., Arain A. A. (2021). Factors influencing the acceptance of mobile collaborative learning for the continuous professional development of teachers. Sustainability.

[B18-behavsci-15-00728] Fornell C., Larcker D. F. (1981). Evaluating structural equation models with unobservable variables and measurement error. Journal of Marketing Research.

[B19-behavsci-15-00728] Gao W. J., Hu Y., Ji J. L., Liu X. Q. (2023). Relationship between depression, smartphone addiction, and sleep among Chinese engineering students during the COVID-19 pandemic. World Journal of Psychiatry.

[B20-behavsci-15-00728] Garg A. (2022). Investigating the moderating effects of age and gender on customers’ use of tablet menu in casual dining restaurants. Journal of Quality Assurance in Hospitality & Tourism.

[B21-behavsci-15-00728] Gerjets P., Scheiter K., Schuh J. (2008). Information comparisons in example-based hypermedia environments: Supporting learners with processing prompts and an interactive comparison tool. Educational Technology Research and Development.

[B22-behavsci-15-00728] Gilster P. (1997). Digital literacy.

[B23-behavsci-15-00728] Greene J. A., Seung B. Y., Copeland D. Z. (2014). Measuring critical components of digital literacy and their relationships with learning. Computers & Education.

[B24-behavsci-15-00728] Hair J. F., Ringle C. M., Sarstedt M. (2011). PLS-SEM: Indeed a silver bullet. Journal of Marketing Theory and Practice.

[B25-behavsci-15-00728] Hair J. F., Risher J. J., Sarstedt M., Ringle C. M. (2019). When to use and how to report the results of PLS-SEM. European Business Review.

[B26-behavsci-15-00728] Hoskins B., Crick R. D. (2010). Competences for learning to learn and active citizenship: Different currencies or two sides of the same coin?. European Journal of Education.

[B27-behavsci-15-00728] Hsu H. P., Wenting Z., Hughes J. E. (2019). Developing elementary students’ digital literacy through augmented reality creation: Insights from a longitudinal analysis of questionnaires, interviews, and projects. Journal of Educational Computing Research.

[B28-behavsci-15-00728] Huang J., Baptista J., Galliers R. D. (2013). Reconceptualizing rhetorical practices in organizations: The impact of social media on internal communications. Information & Management.

[B29-behavsci-15-00728] Jang M., Aavakare M., Nikou S., Kim S. (2021). The impact of literacy on intention to use digital technology for learning: A comparative study of Korea and Finland. Telecommunications Policy.

[B30-behavsci-15-00728] Kennedy D. M., Fox R. (2013). ‘Digital natives’: An Asian perspective for using learning technologies. International Journal of Education and Development Using ICT.

[B31-behavsci-15-00728] Kim H. J., Hong A. J., Song H. D. (2019). The roles of academic engagement and digital readiness in students’ achievements in university e-learning environments. International Journal of Educational Technology in Higher Education.

[B32-behavsci-15-00728] Küsel J., Martin F., Markic S. (2020). University students’ readiness for using digital media and online learning—Comparison between Germany and the USA. Education Sciences.

[B33-behavsci-15-00728] Lankshear C., Knobel M. (2015). Digital literacy and Digital Literacies:-policy, pedagogy and research considerations for education. Nordic Journal of Digital Literacy.

[B34-behavsci-15-00728] Le B., Lawrie G. A., Wang J. T. (2022). Student self-perception on digital literacy in STEM blended learning environments. Journal of Science Education and Technology.

[B35-behavsci-15-00728] Le T. L. H., Hoang V. H., Hoang M. D. M., Nguyen H. P., Bui X. B. (2022). Impact of digital literacy on intention to use technology for online distribution of higher education in Vietnam: A study of Covid19 context. Journal of Distribution Science.

[B36-behavsci-15-00728] Lilian A. (2022). Motivational beliefs, an important contrivance in elevating digital literacy among university students. Heliyon.

[B37-behavsci-15-00728] Lim J., Newby T. J. (2021). Preservice teachers’ attitudes toward Web 2.0 personal learning environments (PLEs): Considering the impact of self-regulation and digital literacy. Education and Information Technologies.

[B38-behavsci-15-00728] Limayem M., Hirt S. G., Cheung C. M. (2007). How habit limits the predictive power of intention: The case of information systems continuance. MIS Quarterly.

[B39-behavsci-15-00728] Liu J., Dai Q., Chen J. (2025). Factors affecting teachers’ use of digital resources for teaching mathematical cultures: An extended UTAUT-2 model. Education and Information Technologies.

[B40-behavsci-15-00728] Liu X., Ji X., Zhang Y., Gao W. (2023). Professional identity and career adaptability among Chinese engineering students: The mediating role of learning engagement. Behavioral Sciences.

[B41-behavsci-15-00728] Makinde O., Okoye M. (2024). How do attitudes towards electronic resources use and information literacy skills predict the use of electronic resources by health sciences postgraduates in Nigeria?. Higher Education Governance and Policy.

[B42-behavsci-15-00728] Miranda J., Navarrete C., Noguez J., Molina-Espinosa J. M., Ramírez-Montoya M. S., Navarro-Tuch S. A., Bustamante-Bello M.-R., Rosas-Fernández J.-B., Molina A. (2021). The core components of education 4.0 in higher education: Three case studies in engineering education. Computers & Electrical Engineering.

[B43-behavsci-15-00728] Mohammadyari S., Singh H. (2015). Understanding the effect of e-learning on individual performance: The role of digital literacy. Computers & Education.

[B44-behavsci-15-00728] Motyl B., Baronio G., Uberti S., Speranza D., Filippi S. (2017). How will change the future engineers’ skills in the Industry 4.0 framework? A questionnaire survey. Procedia Manufacturing.

[B45-behavsci-15-00728] Muasyaroh H., Royanto L. R. (2024). Digital literacy, attitudes toward e-learning, and task value roles in college students’ distance learning self-regulation. Psychological Research on Urban Society.

[B46-behavsci-15-00728] Nikolopoulou K., Gialamas V., Lavidas K. (2020). Acceptance of mobile phone by university students for their studies: An investigation applying UTAUT2 model. Education and Information Technologies.

[B47-behavsci-15-00728] Pham L., Limbu Y. B., Bui T. K., Nguyen H. T., Pham H. T. (2019). Does e-learning service quality influence e-learning student satisfaction and loyalty? Evidence from Vietnam. International Journal of Educational Technology in Higher Education.

[B48-behavsci-15-00728] Potnis D., Deosthali K., Zhu X., McCusker R. (2018). Factors influencing undergraduate use of e-books: A mixed methods study. Library & Information Science Research.

[B49-behavsci-15-00728] Rafi M., JianMing Z., Ahmad K. (2019). Technology integration for students’ information and digital literacy education in academic libraries. Information Discovery and Delivery.

[B50-behavsci-15-00728] Ramírez-Donoso L., Perez-Sanagustin M., Neyem A., Alario-Hoyos C., Hilliger I., Rojos F. (2023). Fostering the use of online learning resources: Results of using a mobile collaboration tool based on gamification in a blended course. Interactive Learning Environments.

[B51-behavsci-15-00728] Reddy P., Chaudhary K., Hussein S. (2023). A digital literacy model to narrow the digital literacy skills gap. Heliyon.

[B52-behavsci-15-00728] Reddy P., Chaudhary K., Sharma B., Chand D. (2021). Contextualized game-based intervention for digital literacy for the Pacific Islands. Education and Information Technologies.

[B53-behavsci-15-00728] Rezeki S. R. I., Dharmawan D., Saksono L., Ekasari S. (2023). Analysis of the influence of digital information quality, technology performance expectancy, technology effort expectancy, price value and social influence on intention to use coffee shop mobile application. Jurnal Informasi dan Teknologi.

[B54-behavsci-15-00728] Risman U., Budiarti A. P. (2023). The influence of performance expectancy, effort expectancy, and social influence on behavioral intention to use ShopeePay. Operations Management and Information System Studies.

[B55-behavsci-15-00728] Saad A. M., Mohamad M. B., Tsong C. K. (2021). Behavioural intention of lecturers towards mobile learning and the moderating effect of digital literacy in Saudi Arabian universities. International Transaction Journal of Engineering, Management, & Applied Sciences & Technologies.

[B56-behavsci-15-00728] Sardone N. B., Devlin-Scherer R. (2010). Teacher candidate responses to digital games: 21st-century skills development. Journal of Research on Technology in Education.

[B57-behavsci-15-00728] Sayaf A. M., Alamri M. M., Alqahtani M. A., Al-Rahmi W. M. (2021). Information and communications technology used in higher education: An empirical study on digital learning as sustainability. Sustainability.

[B58-behavsci-15-00728] Shehzadi S., Nisar Q. A., Hussain M. S., Basheer M. F., Hameed W. U., Chaudhry N. I. (2021). The role of digital learning toward students’ satisfaction and university brand image at educational institutes of Pakistan: A post-effect of COVID-19. Asian Education and Development Studies.

[B59-behavsci-15-00728] Skulmowski A., Xu K. M. (2022). Understanding cognitive load in digital and online learning: A new perspective on extraneous cognitive load. Educational Psychology Review.

[B60-behavsci-15-00728] Songkram N., Chootongchai S., Osuwan H., Chuppunnarat Y., Songkram N. (2023). Students’ adoption towards behavioral intention of digital learning platform. Education and Information Technologies.

[B61-behavsci-15-00728] Šumak B., Šorgo A. (2016). The acceptance and use of interactive whiteboards among teachers: Differences in UTAUT determinants between pre-and post-adopters. Computers in Human Behavior.

[B62-behavsci-15-00728] Tam C., Santos D., Oliveira T. (2020). Exploring the influential factors of continuance intention to use mobile Apps: Extending the expectation confirmation model. Information Systems Frontiers.

[B63-behavsci-15-00728] Tang C. M., Chaw L. Y. (2016). Digital Literacy: A prerequisite for effective learning in a blended learning environment?. Electronic Journal of E-Learning.

[B64-behavsci-15-00728] Tarhini A., Masa’deh R. E., Al-Busaidi K. A., Mohammed A. B., Maqableh M. (2017). Factors influencing students’ adoption of e-learning: A structural equation modeling approach. Journal of International Education in Business.

[B65-behavsci-15-00728] Tejedor S., Cervi L., Pérez-Escoda A., Jumbo F. T. (2020). Digital literacy and higher education during COVID-19 lockdown: Spain, Italy, and Ecuador. Publications.

[B66-behavsci-15-00728] Ting Y. L. (2015). Tapping into students’ digital literacy and designing negotiated learning to promote learner autonomy. The Internet and Higher Education.

[B67-behavsci-15-00728] Tseng T. H., Lin S., Wang Y. S., Liu H. X. (2022). Investigating teachers’ adoption of MOOCs: The perspective of UTAUT2. Interactive Learning Environments.

[B68-behavsci-15-00728] Venkatesh V., Morris M. G., Davis G. B., Davis F. D. (2003). User acceptance of information technology: Toward a unified view. MIS Quarterly.

[B69-behavsci-15-00728] Venkatesh V., Thong J. Y., Xu X. (2012). Consumer acceptance and use of information technology: Extending the unified theory of acceptance and use of technology. MIS Quarterly.

[B70-behavsci-15-00728] Venkatesh V., Thong J. Y., Xu X. (2016). Unified theory of acceptance and use of technology: A synthesis and the road ahead. Journal of the Association for Information Systems.

[B71-behavsci-15-00728] Vodă A. I., Cautisanu C., Grădinaru C., Tănăsescu C., de Moraes G. H. S. M. (2022). Exploring digital literacy skills in social sciences and humanities students. Sustainability.

[B72-behavsci-15-00728] Wijaya T. T., Su M., Cao Y., Weinhandl R., Houghton T. (2025). Examining Chinese preservice mathematics teachers’ adoption of AI chatbots for learning: Unpacking perspectives through the UTAUT2 model. Education and Information Technologies.

[B73-behavsci-15-00728] Yustika G. P., Iswati S. (2020). Digital literacy in formal online education: A short review. Dinamika Pendidikan.

[B74-behavsci-15-00728] Zhou Y., Li X., Wijaya T. T. (2022). Determinants of behavioral intention and use of interactive whiteboard by K-12 teachers in remote and rural areas. Frontiers in Psychology.

[B75-behavsci-15-00728] Zhu T., Yang Y. (2023). Research on mobile learning platform interface design based on college students’ visual attention characteristics. PLoS ONE.

